# Risk of low bone mineral density in patients with rheumatoid arthritis treated with biologics

**DOI:** 10.1186/s12891-015-0732-x

**Published:** 2015-09-30

**Authors:** Kengo Takahashi, Takao Setoguchi, Hiroki Tawaratsumida, Yoshiya Arishima, Hiroyuki Tominaga, Yasuhiro Ishidou, Satoshi Nagano, Sanae Shigemizu, Noriko Aoki, Masaki Akimoto, Hideo Otsubo, Takemasa Matsuda, Hironori Kakoi, Toshihiko Izumi, Shunsuke Nakamura, Masahiro Yokouchi, Nobuhiko Sunahara, Setsuro Komiya

**Affiliations:** Department of Orthopaedic Surgery, Graduate School of Medical and Dental Sciences, Kagoshima University, Kagoshima, Japan; Japanese Red Cross Kagoshima Hospital, Kagoshima, Japan; The Near-Future Locomotor Organ Medicine Creation Course (Kusunoki Kai), Graduate School of Medical and Dental Sciences, Kagoshima University, 8-35-1 Sakuragaoka, Kagoshima, 890-8520 Japan; Department of Medical Joint Materials, Graduate School of Medical and Dental Sciences, Kagoshima University, Kagoshima, Japan

**Keywords:** Biologics, Osteoporosis, Rheumatoid arthritis (RA), Risk factors, Young adult mean (YAM)

## Abstract

**Background:**

Osteoporosis is a complication of rheumatoid arthritis (RA). We identified risk factors for osteoporosis during treatment with biologics.

**Methods:**

Femoral neck bone mineral density (BMD) was measured in 186 patients with biologics-treated RA. We compared the characteristics of those with BMD ≥70 % of young adult mean (YAM) and those with BMD <70 % of YAM, and undertook multivariable logistic regression analysis to identify risk factors for bone loss.

**Results:**

Mean age and disease duration, the proportion of females, scores in the Modified Health Assessment Questionnaire and history of vertebral fracture were significantly greater in the BMD <70 % of YAM group, but body mass index (BMI) was significantly lower in the BMD <70 % of YAM group. There was no significant difference between the groups in terms of other biomarkers of RA activity, the proportion treated with methylprednisolone, or the duration or choice of biologics. The proportions of patients treated with anti-osteoporosis drugs and parathyroid hormone were significantly higher in the BMD <70 % of YAM group. In the multivariable analysis, advanced age, female, longer disease duration, history of past thoracic or lumbar vertebral fracture, higher Steinbrocker classification and lower BMI were significant factors for BMD <70 % of YAM.

**Discussion:**

We identified risk factors for bone loss in patients with RA treated with biologics. Before suppression of disease activity by biologics, bone loss might already be advanced.

**Conclusions:**

We recommend that patients with RA who possess these risk factors be considered for earlier and more intense treatment to prevent bone loss, as well as addressing RA disease progression.

**Electronic supplementary material:**

The online version of this article (doi:10.1186/s12891-015-0732-x) contains supplementary material, which is available to authorized users.

## Background

Rheumatoid arthritis (RA) is a systemic inflammatory disease that can cause local joint deformity, including erosions of bone and narrowing of the joint space, and extra-articular symptoms, including anemia, pneumonitis and osteoporosis. Osteoporosis increases the risk of fracture and causes pain and disability, impairing the quality of life of patients with RA. High disease activity, glucocorticoid therapy, immobility, advanced age, low body mass and female sex are reportedly risk factors for osteoporosis in patients with RA [1]. Inflammation is the one of the key triggers of bone resorption and contributes to local and generalized osteoporosis [2]. Inflammatory cytokines activate osteoclast differentiation, which resorb bone matrix. Osteoclasts play a central role in bone resorption in RA, orchestrated by T-lymphocytes, monocytes and fibroblasts in the synovium of inflammatory joints, which produce osteoclast differentiation-inducing factors. Osteoclast differentiation is mainly promoted by the receptor activator of nuclear factor-kappa B ligand (RANKL), which is up-regulated by a large number of the inflammatory cytokines involved in the pathogenesis of RA [2]. A better understanding of the pathogenesis of RA has improved treatment of the disease, particularly the targeting of key molecules by biologics [3–5]. Beyond the control of RA activity, there is evidence that biologics might also have beneficial effects on bone metabolism and bone remodeling [6]. We examined the bone mineral density (BMD) of patients with RA treated with biologics, and aimed to establish which factors were associated with low BMD.

## Methods

### Patients

We retrospectively studied the records of 186 consecutive patients with RA diagnosed using the 2010 criteria of the American College of Rheumatology and treated at the Japanese Red Cross Kagoshima Hospital with infliximab, adalimumab, golimumab, etanercept, tocilizumab or abatacept according to established protocols.

### BMD of the femoral neck

Bone mineral density was measured between December 2011 and December 2013 using the Discovery DXA system (Hologic, Waltham, Massachusetts, USA). The BMD of the femoral neck (in g/cm^2^) was calculated using the young adult mean (YAM). The Japanese Society for Bone and Mineral Research has proposed that primary osteoporosis should be diagnosed when BMD is <80 % of YAM with evidence of a fragility fracture, or when BMD is <70 % of YAM [7–10]. The patients were divided into two groups: those with BMD <70 % of YAM and those with BMD ≥70 % of YAM.

### Demographic and disease-related data

Patients’ demographic and clinical characteristics were recorded from their medical records, including BMD, age, sex, body mass index (BMI), disease duration, presence or absence of rheumatoid vasculitis [11], dose of methylprednisolone, serum C-reactive protein (CRP) concentration, duration and type of biologics administered, anti-osteoporosis drugs used, Disease Activity Score 28-CRP (DAS28-CRP), Simplified Disease Activity Index (SDAI), Clinical Disease Activity Index (CDAI), Modified Health Assessment Questionnaire (MHAQ) score, the Steinbrocker criteria, and presence or absence of previous vertebral or femoral neck fracture.

### Statistical analysis

The Kolmogorov–Smirnov test was performed to examine the distribution of data, and data were evaluated using the independent Student’s *t* test, Mann–Whitney *U* test, Fisher’s exact test, Pearson’s chi-square test, or Kruskal–Wallis test as appropriate, using Excel Statistics 2012 and Excel Statistics 2015 (SSRI, Tokyo, Japan). P values <0.05 were considered statistically significant. Baseline factors were examined by univariate analysis for the BMD ≥70 % of YAM group. P-values <0.2 were further examined by Spearman’s correlation coefficient to identify confounding factors. Multivariable logistic regression analysis included age, sex, disease duration, history of past vertebral fracture, BMI, MHAQ score, Steinbrocker classification, and duration of bDMARD use. Multiple logistic regression was performed to select the best model for predicting risk factors associated with a BMD <70 % of YAM using SPSS software (Kondo Photo Process Co., Ltd., Osaka, Japan).

### Ethics statement

The Ethics Committee on Clinical Research at the Japanese Red Cross Kagoshima Hospital approved the research protocol.

### Consent statement

All patients gave written informed consent for their data to be used in the study.

## Results

### Comparison of BMD <70 % of YAM and BMD ≥70 % of YAM groups

Of the 186 patients who underwent DEXA scanning of the femoral neck, 57 had BMD <70 % of YAM and 129 had BMD ≥70 % of YAM. Age was significantly higher in the BMD <70 % of YAM group (median 65.0 years) than the BMD ≥70 % of YAM group (median 58.0 years). The proportion of women was significantly higher in the BMD <70 % of YAM group (*p* = 0.008). Disease duration was significantly longer in the BMD <70 % of YAM group. Body mass index was significantly lower in the BMD <70 % of YAM group (Table 1).

**Table 1:** Demographic and clinical characteristics of the BMD <70 % of YAM and BMD ≥70 % of YAM groups

	BMD <70 % of YAM	BMD ≥70 % of YAM	*p* value
Age	65.0 (58.0–69.0)	58.0 (53.0–66.0)	0.0044*
Proportion female	93.0 %	77.9 %	0.0079*
Disease duration (year)	15.0 (8.0–20.0)	8.0 (4.0–15.0)	0.0004*
BMI	21.4 ± 2.8	23.6 ± 3.3	<0.0001*
Rate of rheumatoid vasculitis	1.8 %	1.6 %	0.67
Proportion taking methylprednisolone	45.6 %	40.3 %	0.52
Dose of methylprednisolone (mg)	2.0 (1.0–3.5)	3.3 (2.0–6.0)	0.65
CRP (mg/dL)	0.09 (0.03–0.31)	0.08 (0.03–0.37)	0.89
DAS28-CRP	2.63 (1.88–3.38)	2.50 (1.70–3.30)	0.49
CDAI	6.70 (2.90–14.90)	5.80 (2.60–12.95)	0.54
SDAI	6.95 (3.05–14.10)	6.30 (2.74–12.94)	0.58
MHAQ score	9.00 (1.00–14.00)	4.00 (0.00–8.00)	0.002*
History of proximal femoral fracture	2/57	0/129	0.087
History of thoracic or lumbar vertebral fracture	17/57	9/129	<0.001*
Duration of biologics therapy	4.2 ± 2.6	3.6 ± 2.3	0.166

*BMD* bone mineral density, *YAM* young adult mean, *BMI* body mass index, *CRP* serum C-reactive protein concentration, *DAS28-CRP* Disease Activity Score-28-CRP, *SDAI* Simplified Disease Activity Index, *CDAI* Clinical Disease Activity Index, *MHAQ* Modified Health Assessment Questionnaire

### Association of methylprednisolone therapy on osteoporosis

We examined whether methylprednisolone therapy influenced the extent of osteoporosis. Twenty-six patients had used methylprednisolone for >3 months in the BMD <70 % of YAM group, compared with 52 in the BMD ≥70 % of YAM group (*p* = 0.52). There was also no significant difference in the daily dose of methylprednisolone between the groups (*p* = 0.65, Table 1).

### Association of disease activity on osteoporosis

We assessed whether there was a relationship between the extent of osteoporosis and biomarkers of disease activity including serum CRP concentration, DAS28, CDAI, SDAI, MHAQ score and the Steinbrocker classification. The MHAQ score was significantly higher in the BMD <70 % of YAM group (median 9) than the BMD ≥70 % of YAM group (median 4, Table 1). The mean serum CRP concentration did not differ significantly between the groups (*p* = 0.89). There were also no significant differences between the groups in terms of DAS28-CRP (*p* = 0.49), CDAI (*p* = 0.54), SDAI (*p* = 0.58, Table 1) or Steinbrocker classification (*p* = 0.11, Table 2).

**Table 2:** Classification of patients according to the Steinbrocker criteria in the BMD <70 % of YAM and BMD ≥70 % of YAM groups

		BMD <70 % of YAM	BMD ≥70 % of YAM
Steinbrocker classification	I	23	69
	II	10	23
	III	21	36
	IV	3	1

(*p* = 0.11)

*BMD* bone mineral density, *YAM* young adult mean

### History of vertebral and femoral neck fractures

Seventeen patients in the BMD <70 % of YAM group had sustained thoracic or lumbar vertebral fracture, compared with nine in the BMD ≥70 % of YAM group (*p* < 0.001), but there was no significant difference in the number of patients who had sustained proximal femoral fracture (*p* = 0.09, Table 1).

### Association of disease activity on osteoporosis of duration or type of biologics therapy on osteoporosis

The duration of biologics therapy did not differ significantly between the groups (*p* = 0.166, Table 1). We classified biologics into: tumor necrosis factor-α (TNFα) inhibitors (infliximab, adalimumab, golimumab and etanercept); tocilizumab; abatacept; and switch biologics (where TNFα inhibitor therapy was switched to tocilizumab or abatacept). There was no significant difference in biologics class between the BMD <70 % of YAM and BMD ≥70 % of YAM groups (*p* = 0.67, Table 3).

**Table 3:** Class of biologics taken in the BMD <70 % of YAM and BMD ≥70 % of YAM groups

	BMD <70 % of YAM	BMD ≥70 % of YAM
TNFα inhibitor	26	66
Tocilizumab	6	16
Abatacept	3	9
Switch biologics	22	38

(*p* = 0.67)

*BMD* bone mineral density, *YAM* young adult mean, *TNF*α tumor necrosis factor-α

### Association of disease activity on osteoporosis of duration or type of biologics therapy on osteoporosis of anti-osteoporosis drug therapy on osteoporosis

The proportion of patients treated with anti-osteoporosis drugs was significantly higher in the BMD <70 % of YAM group (*p* = 0.004, Table 4). Having divided the anti-osteoporosis drugs into bisphosphonates, parathyroid hormone (PTH) and others (such as vitamin D and raloxifene), we found that the proportion of patients treated with PTH was significantly higher in the BMD <70 % of YAM group (*p* = 0.008, Table 5).

**Table 4:** Use of anti-osteoporosis drugs in the BMD <70 % of YAM and BMD ≥70 % of YAM groups

	BMD <70 % of YAM	BMD ≥70 % of YAM
None	13	58
Anti-osteoporosis drug use (total)	44	71

(*p* = 0.004)

*BMD* bone mineral density, *YAM* young adult mean

### Multiple logistic regression analysis of factors associated with BMD <70 % of YAM

We performed univariate analysis (Additional file 1: Table S1) and multiple logistic regression analysis of factors associated with a BMD <70 % of YAM. Spearman’s correlation coefficient revealed no high relationships between a BMD <70 % of YAM and age, sex, disease duration, history of vertebral fracture, BMI, MHAQ, Steinbrocker classification, or duration of biologics use. Multiple logistic regression analysis showed that age (odds ratio [OR] 1.065), female (OR 5.019), disease duration (OR 1.077), history of vertebral fracture (OR 7.708), and Steinbrocker classification (OR 2.302) were associated with a greater risk of a BMD <70 % of YAM, whereas higher BMI (OR 0.766) reduced the risk for a BMD <70 % of YAM (Table 6).

**Table 5:** Type of anti-osteoporosis drugs used in the BMD <70 % of YAM and BMD ≥70 % of YAM groups

	BMD <70 % of YAM	BMD ≥70 % of YAM	p value
Bisphosphonate	30	54	0.087
PTH	4	1	0.008*
Others	10	16	0.351

*BMD* bone mineral density, *YAM* young adult mean

**Table 6:** Multiple logistic regression analysis of factors associated with bone mineral density <70 % of young adult mean

Feature	Odds ratio	95 % confidence intervals	*p* value
Age (per 1 year increase)	1.065	1.015–1.101	0.003*
sex	5.019	1.367–18.43	0.015*
Disease duration (per 1 year increase)	1.077	1.028–1.128	0.002*
History of past vertebral fracture	7.708	2.505–23.72	<0.001*
BMI (per 1 point increase)	0.766	0.665–0.883	<0.001*
Steinbrocker classification	2.302	1.473–3.597	<0.001*

*BMD* bone mineral density, *BMI* body mass index

## Discussion

The Japanese Society for Bone and Mineral Research proposed revised diagnostic criteria for primary osteoporosis in 2012. Primary osteoporosis is diagnosed when BMD is <80 % of YAM with evidence of a fragility fracture, or <70 % of YAM [7–9]. We therefore elected to identify risk factors for a BMD <70 % of YAM.

Although the presence of osteoporosis, as identified by DEXA, is the best predictor of fracture in patients with RA [12], many indices of risk have been reported to identify low BMD, including the Simple Calculated Osteoporosis Risk Estimation (SCORE), the Osteoporosis Risk Assessment Instrument (ORAI), the Osteoporosis Self-Assessment Tool, the Osteoporosis Index of Risk (OSIRIS) and the Fracture Risk Assessment Tool (FRAX). Although these indices have substantial sensitivity in populations not affected by RA, they do not satisfactorily predict low BMD in RA [13]. We showed that age, female sex, disease duration, history of vertebral fracture, Steinbrocker classification, and lower BMI were associated with a greater risk of bone loss in patients with RA treated by biologics. There is still substantial debate about the risk factors for osteoporosis in patients with RA. The erythrocyte sedimentation rate, DAS, corticosteroid therapy, anti-resorptive osteoporosis treatment, MHAQ score, swollen joint count, tender joint count, hormone replacement therapy, disease severity, BMI, immobilization, and disease duration have all been implicated [14–18]. Disease duration and disease activity has been considered the most important risk factors for osteoporosis [2, 19, 20]. Although our risk factors were found in patients with RA treated with biologics, these risk factors are compatible with those in the general population [21–23]. We also found that methylprednisolone therapy, serum CRP concentration, DAS28-CRP, CDAI and SDAI were not risk factors for low BMD. These discrepancies may be a consequence of treatment with biologics. Before suppression of disease activity by biologics, bone loss might already be advanced. Bone loss is often found in patients with recent-onset RA [24] and bone loss has reportedly already started during the autoimmune phase of RA, long before inflammation occurs [25], implying that therapy for osteoporosis should be initiated promptly. Further research will be needed to establish whether these factors could be used prospectively as predictors of osteoporosis and fracture in patients with RA. We found only one English-language paper in PubMed that showed the relationship between the Steinbrocker classification and bone loss in patients with RA. The article reported that a higher Steinbrocker classification is a risk factor for bone loss of the femoral neck and lumbar spine [26]. Our findings are compatible with this report.

As well as influencing disease activity, biologics may prevent bone loss via a direct effect on bone metabolism. It is well recognized that TNFα induces differentiation of osteoclast precursors through a synergistic action with RANKL [27]. Bone metabolism and remodeling are regulated by a balance between TNF superfamily molecules, RANKL, osteoprotegerin, osteoclastogenesis inhibitory factor and TNF-related apoptosis-inducing ligand [12, 28, 29]. These cytokines are responsible for the imbalance between bone resorption and formation in RA, which may explain the ability of biologics not only to suppress systemic inflammation, but also to prevent bone loss [30–33]. Nonetheless, there appears to be no significant difference in BMD change in biologics responders and non-responders [34, 35]. In addition, we found that the class and duration of biologics therapy was not significantly different in the BMD <70 % of YAM and BMD ≥70 % of YAM groups. Sequential evaluation of BMD during biologics therapy should help to illuminate their influence on bone density.

Although the proportion of patients treated with anti-osteoporosis drugs was significantly higher in the BMD <70 % of YAM group, bone loss could not be completely prevented. In addition, the proportion of patients treated with PTH was significantly higher in the BMD <70 % of YAM group, even though the absolute number of patients was only four. These findings suggest that patients who possess the risk factors that we have identified require earlier and more intensive treatment to prevent bone loss.

Our study has some limitations. First, data collection was retrospective. Second, BMD was measured once in each patient, so longitudinal data are not available. Third, we did not measure the change in biomarkers of bone remodeling in the blood or urine. Although there is reportedly no change in the indices of bone remodeling after 1 year of biologics treatment [30, 35], we are now examining longitudinal changes in BMD and biomarkers of bone turnover in a prospective study.

## Conclusions

We identified risk factors for bone loss in patients with RA treated with biologics. As fragility bone fracture may substantially impair quality of life, and also has substantial adverse socioeconomic consequences, our findings suggest that osteoporosis should be detected and addressed promptly in patients with bDMARD-treated RA who possess these risk factors.

## Additional file

Additional file 1: Table S1. Univariate analysis of factors potentially associated with low bone mineral density in patients with rheumatoid arthritis treated with biologics. (DOCX 14 kb)

## Abbreviations

RA: Rheumatoid arthritis
YAM: Young adult mean
RANKL: Receptor activator of nuclear factor-kappa B ligand
BMD: Bone mineral density
DEXA: Dual X-ray absorptiometry
BMI: Body mass index serum
CRP: C-reactive protein
DAS28-CRP: Disease activity score 28 CRP
SDAI: Simplified disease activity index
CDAI: Clinical disease activity index
MHAQ: Modified health assessment questionnaire
IQR: Interquartile range
OR: Odds ratio
CI: Confidence interval
TNFα: Tumor necrosis factor-alpha
PTH: Parathyroid hormone
SCORE: Calculated osteoporosis risk estimation
ORAI: Osteoporosis risk assessment instrument osteoporosis self-assessment tool
OSIRIS: Osteoporosis index of risk
FRAX: Fracture risk assessment tool
