# Supplementary material for: Risk of low bone mineral density in patients with rheumatoid arthritis treated with biologics
Source: BMC Musculoskelet Disord. 2015 Sep 30;16:269. doi: 10.1186/s12891-015-0732-x (PMC4589107; doi:10.1186/s12891-015-0732-x)
Supplement: Additional file 1: Table S1. — Univariate analysis of factors potentially associated with low bone mineral density in patients with rheumatoid arthritis treated with biologics. (DOCX 14 kb) [file 12891_2015_732_MOESM1_ESM.docx]

Additional file 1: Table S1: Univariate analysis of factors potentially associated with low bone mineral density in patients with rheumatoid arthritis treated with biologics

|  | Univariate relative odds | 95% confidence interval | p value |
| --- | --- | --- | --- |
| Age | 1.047 | 1.014-1.081 | 0.005 † |
| Proportion of females | 3.507 | 1.166-10.550 | 0.026 † |
| Disease duration (year) | 1.073 | 1.032-1.115 | <0.001 † |
| BMI | 0.789 | 0.703-0.887 | 0.001 † |
| Rheumatoid vasculitis | 1.134 | 0.101-12.764 | 0.919 |
| Methylprednisolone use | 1.333 | 0.711-2.500 | 0.370 |
| Dose of methylprednisolone (mg) | 0.966 | 0.848-1.100 | 0.596 |
| CRP (mg/dL) | 0.978 | 0.597-1.602 | 0.928 |
| DAS28-CRP | 1.091 | 0.842-1.413 | 0.509 |
| CDAI | 1.009 | 0.977-1.041 | 0.592 |
| SDAI | 1.008 | 0.976-1.040 | 0.639 |
| MHAQ score | 1.091 | 1.035-1.0151 | 0.001 † |
| History of thoracic or lumbar vertebral fracture | 5.667 | 2.342-13.712 | <0.001 † |
| Steinbrocker classification | 1.425 | 1.021-1.990 | 0.038 † |
| Duration of biologics use | 1.107 | 0.974-1.259 | 0.121 |
| Steroid use (more than 3 months) | 1.077 | 0.572-2.027 | 0.818 |

Table legend:

Abbreviations: BMD, bone mineral density; YAM, young adult mean; BMI, body mass index; CRP, serum C-reactive protein concentration; DAS28-CRP, Disease Activity Score-28-CRP; SDAI, Simplified Disease Activity Index; CDAI, Clinical Disease Activity Index; MHAQ, Modified Health Assessment Questionnaire.
